# Versatile biocatalyst: lipase from *Streptomyces gobitricini* for ester synthesis and detergent innovation

**DOI:** 10.3389/fbioe.2025.1589087

**Published:** 2025-05-16

**Authors:** Areej Ali Alzahrani, Najeh Krayem, Mona Alonazi, Jihan M. Al-Ghamdi, Habib Horchani, Abir Ben Bacha

**Affiliations:** ^1^ Department of Biochemistry, College of Science, King Saud University, Riyadh, Saudi Arabia; ^2^ Laboratory of Biochemistry and Enzymatic Engineering of Lipases, ENIS, University of Sfax, Sfax, Tunisia; ^3^ Science Department, Environmental and biotechnology research group, College of Rivière-Du-Loup, Quebec, QC, Canada

**Keywords:** industrial applications, biocatalysis, lipase purification, stability, detergent formulations, esterification

## Abstract

**Background/objectives:**

The growing demand for reliable and stable biocatalysts has spurred research into microbial lipases for diverse industrial applications. This study focused on enhancing the production and purification of a lipase from *Streptomyces gobitricini* (Lip_S.g_).

**Methods:**

Maximal lipase activity (420 U/mL) was achieved during the stationary phase after 84 h of incubation at 45°C and pH 8.0, using 2% glucose and 2% yeast extract as carbon and nitrogen sources, respectively.

**Results:**

Calcium, olive oil, and Tween, at 1%, significantly enhanced Lip_S.g_ production, highlighting the role of triglycerides and detergents in enzyme induction and substrate emulsification. The purified 50-kDa enzyme displayed maximal activity at 50°C and pH 9.0, with thermal stability between 40°C and 55°C and pH 5.0–10.0. While Lip_S.g_ efficiently hydrolyzed short and medium-chain triglycerides, it exhibited a preference for long-chain substrates, with a maximum reaction rate of 2500 μmol/min/mg and a K_m_ value of 6.45 mM toward triolein (C18). Lip_S.g_ also demonstrated remarkable stability in detergent formulations, retaining more than 85% activity in the presence of surfactants, oxidizing agents, boron compounds, and enzyme inhibitors. Additionally, Lip_S.g_ catalyzed the esterification of oleic acid with starch and ethanol to produce starch oleate and ricinoleic acid.

**Conclusion:**

These findings establish Lip_S.g_ as a promising biocatalyst for applications in biocatalysis and detergent formulations, with potential uses in the food, beverage, cosmetic, and pharmaceutical industries.

## 1 Introduction

Lipases (EC 3.1.1.3) are triacylglycerol acyl-hydrolases that hydrolyze triglycerides at the organic-aqueous interface, producing diglycerides, monoglycerides, glycerol, and free fatty acids (Adetunji and Olaniran, 2021). These enzymes are serine hydrolases with a catalytic triad of Serine, Aspartate/Glutamate, and Histidine (Gupta et al., 2004). Their molecular weight is in the range of 19–60 kDa and reported as monomeric proteins (Chandra et al., 2020; Abdelaziz et al., 2025). Lipases are classified based on their substrate specificity into non-specific, 1, 3-specific, and fatty acid-specific types (Yao et al., 2021). Non-specific lipases hydrolyze triglycerides into free fatty acids and glycerol. 1, 3-specific lipases act on the ester bonds at the C1 and C3 positions of triglycerides. Fatty acid-specific lipases preferentially hydrolyze long-chain fatty acids with cis-double bonds at the C9-C10 position, like triolein (Yao et al., 2021).

Lipases are widespread in nature and are produced by a diverse range of organisms, including plants, animals, and microorganisms (Bharathi and Rajalakshmi, 2019). The majority of microbial lipases are secreted as extracellular enzymes, making them relatively easy to isolate in high purity and ideal for large-scale production (Yao et al., 2021). Microbial lipases are preferred compared to those from animals and plants due to the rapid growth and reproduction rates of microbes facilitating the production of these enzymes with high yields (Yao et al., 2021). Among microorganisms, lipase production is especially prevalent in bacteria, fungi, and yeast. The most common bacterial sources of lipases include species from *Bacillus*, *Pseudomonas, Staphylococcus*, and *Burkholderia* (Bharathi and Rajalakshmi, 2019).

Bacterial lipase production is strongly affected by physicochemical conditions of medium culture, including temperature, pH, agitation speed, nitrogen and carbon sources, inorganic salts as sources of metal ions and dissolved oxygen levels (Gupta et al., 2004). Oils, triacylglycerols, fatty acids, hydrolysable esters, tweens, bile salts and glycerol are usually opadded in growth bacterial medium to induce the lipase production since they are inducible enzymes. Moreover, sugar alcohol, polysaccharides or whey as well as long-chain fatty acids, such as oleic, linoleic and linolenic acids were described to enhance lipase production (Gupta et al., 2004; Cesário et al., 2021; Al Mohaini et al., 2022). Inorganic and organic nitrogen sources, have proven effective for lipase production in some microorganisms. Likewise, divalent cations especially calcium (Ca^2+^) and magnesium (Mg^2+^) enhanced efficiency the bacterial lipase yield production (Gupta et al., 2004). Reported bacterial lipases was overexpressed generally at pH 7.0–8.0, temperature growth between 20°C and 50°C during an incubation period varying from 24 h to 96 h (Gupta et al., 2004). Only a limited number of media have been described for enhancing growth conditions of *Streptomyces* strains to produce extracellular lipases. For instance, *Streptomyces* sp. *CS326* was cultivated at 28°C for 168 h in a medium containing 10 g of glucose, 10 g of soybean, and 0.1 g of Na_2_HPO_4_. *Streptomyces halstedii* strain ST 40 showed enhanced lipase activity when incubated with Tween 20 for 4 h. Maximal lipase production from *Streptomyces griseus* was achieved using sunflower oil and palm oil in an orbital shaker after 96 h (Vasconcelos, 2018).

Microbial lipases have been extensively studied due to their diverse biochemical properties and industrial applications. Typically, their maximal pH is neutral or alkaline, with maximal temperature etween 30°C and 60°C. Bacterial lipases exhibit stability across a wide pH range (pH 4.0–11.0) (Yuan et al., 2016). They are regiospecific, often 1, 3-specific, selectively hydrolyzing the ester bonds at the C1 and C3 positions of glycerol. This process breaks down triacylglycerides into free fatty acids, 1, 2(2,3)-diacylglycerides, and 2-monoacylglycerides (Gupta et al., 2004). At organic-aqueous interfaces, microbial lipases are activated, and surfactants can either enhance or inhibit their activity based on interactions and enzyme-surfactant complex formation (Delorme et al., 2011).

Due to their versatile enzymatic properties, substrate specificity, and high stability, microbial lipases are a valuable class of biotechnological enzymes used in diverse applications across industries, including food, leather, pharmaceuticals, textiles, cosmetics, and especially detergents (Adetunji and Olaniran, 2021). Furthermore, using lipases as a detergent additive is the most important industrial application (Moayd and Yan, 2017; Reyes-Reyes et al., 2022; Eskandari et al., 2024). As reported by the Brainy Insights, a company specializing in global and regional market research, the global microbial lipase market was valued at USD 518.40 million in 2021. This market is projected to grow from 2022 to 2030, reaching an estimated USD 898.40 million by 2030 (Rodríguez-Alonso et al., 2023). Detergent enzymes must be active in alkaline conditions and high temperatures, and stable in complex mixtures with Ca^2+^ ions, surfactants, oxidizers, and proteases. Lipases are suitable due to their broad substrate specificity, resilience in harsh washing conditions, and ability to remain active with various detergent components (Adetunji and Olaniran, 2021; Guncheva and Zhiryakova, 2011). Lipases in detergents effectively break down oily stains, reducing the need for harsh chemicals. They are also eco-friendly, leaving no toxic residues and being safe for aquatic ecosystems (Adetunji and Olaniran, 2021). Lipolase, derived from *Thermomyces lanuginosus*, was the first industrial lipase introduced into detergents, launched in 1988 by Novo Nordisk. Other lipases, such as Lumafast from *Pseudomonas mendocina* and Lipomax from *Pseudomonas alcaligenes*, were later commercialized by Genencor (Yao et al., 2021). The common source strains of lipase used in the detergent industry include *Bacillus*, *Geobacillus*, *Pseudomonas*, and *Serratia* genera (Chandra et al., 2020). Even though many lipases from different species are found in the literature, the enzyme market is still growing and the need for research of new enzymes remains necessary for potential biotechnological applications.

The production of low molecular weight flavor esters is crucial in food industry, where lipases serve as additives for various flavors and perfumes (Mehta et al., 2021). For instance, lipases from *Bacillus aerius* and *Geobacillus sp*., have been used to synthesize isoamyl acetate and methyl salicylate, respectively, both of which enhance the flavor of confectionery products such as chewing gums (Narwal and Gupta, 2013). Lipases were generally used as biocatalysts to enhance flavors and modify their structures through inter- or trans-esterification. Likewise, they modify flavor by synthesizing esters from short-chain fatty acids and alcohols, which are recognized as flavor and fragrance compounds (Mehta et al., 2021).

*Streptomyces* genus are among the most prolific producers of secondary metabolites, contributing to a significant portion of naturally derived bioactive compounds used in medicine today (Lacey and Rutledge, 2022). Historically, *Streptomyces* strains have been the primary source of the largest number of new antibiotic drugs as secondary metabolites compared to both bacteria and fungi. This genus represents a potent source of antibiotic including tetracycline, chloramphenicol, erythromycin, and aminoglycosides as well as quinine antibiotics (Taher et al., 2020). Additionally, *Streptomyces* spp. Produce a variety of extracellular enzymes especially xylanase, chitinase, and cellulase that play a crucial role in breaking down biomass into assimilable carbon units and are widely utilized in industrial and agricultural applications due to their significant large-scale fermentation capacity and proficiency in bulk manufacturing (Vasconcelos, 2018). While researchers are isolating novel *Streptomyces* strains from unexplored habitats to assess their secreted products potential and effectiveness, studies on *Streptomyces* lipases remain limited compared to those of other bacterial strains (Spasic et al., 2018). Despite the increasing number of studies in this area, further research is still needed to fully discover and understand lipase production and activity within the *Streptomyces* genus. As the demand for detergent-compatible enzymes and for esters production rises, ongoing efforts are being made to screen new bacteria capable of producing lipases with enhanced stability. This study focused on refining the culture conditions, purifying, and biochemically characterizing a novel thermostable lipase from *Streptomyces gobitricini* (*S. gobitricini*) strain. Additionally, the significant potential of this new lipase for industrial applications, particularly in detergent formulations and ester production, has been evaluated.

## 2 Results

### 2.1 Effect of medium components on Lip_S.g_ production

#### 2.1.1 Incubation time

**FIGURE 1:**
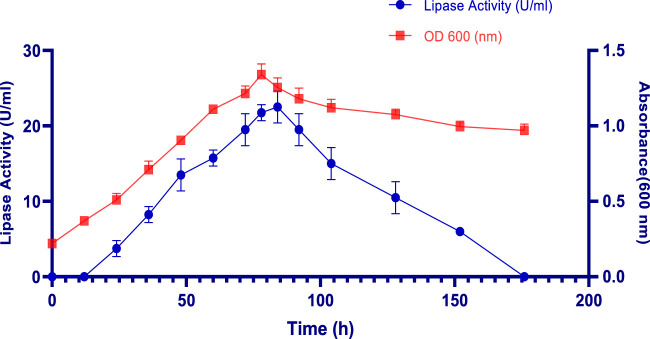
Effect of incubation time on S. gobitricini cell growth and lipase production. Results were presented as mean ± SD from three independent experiments.

The effect of incubation time on Lip_S.g_ production was monitored over 176 h, as shown in Figure 1. During the initial 24 h, a notable increase in absorbance was observed, indicating the exponential growth phase, where *S. gobitricini* focused on rapid cell division and biomass accumulation rather than lipase production, a pattern common to many *Streptomyces* species (Ousaadi et al., 2021). Lipase activity started to rise significantly after 48 h, peaking at 84 h with an absorbance of 1.25 ± 0.06 (Figure 1). The production of lipase, reaching 22.50 ± 2.12 U/mL corresponding to 58925,5 U/g, was closely tied to cell growth during the stationary phase, when nutrient depletion and stress signals triggered the production of secondary metabolites like extracellular lipases (Bhatt et al., 2020). This increase was also attributed to the bacteria’s need to scavenge lipids as alternative carbon sources under nutrient-limited conditions. After 84 h, enzymatic activity declined gradually, falling to 6 U/mL (28284,2 U/g) by 152 h (Figure 1). The biomass, measured by absorbance at 600 nm, gradually declined between 84 and 176 h, reaching approximately 0.97 ± 0.042. This decrease is likely due to autolysis, as cells undergo breakdown in response to nutrient depletion and waste accumulation a typical process during the bacterial decline phase. This reduction in biomass also corresponded with decreased enzymatic activity, reflecting a loss of cell viability (Ousaadi et al., 2021). Similar findings were observed with *Streptomyces* sp. Al-Dhabi-49, isolated from Saudi Arabian soil, where lipase activity was initially detected at 24 h, peaking at 253 ± 4.4 U/mL after 5 days, before slightly declining to 172 ± 2.1 U/mL after 6 days 23 (Al-Dhabi et al., 2020). Additionally, *Streptomyces sp*. A3301 produced a thermostable lipase with an enzymatic activity of 108 U/mL after 168 h of incubation in the production medium (Al-Dhabi et al., 2020). Likewise, recombinant lipase from *Streptomyces bacillaris* expressed in *Bacillus subtilis* reached its maximal production after 12H fermentation (Gao et al., 2021). A potential thermostable lipase (designated MAS1) from the marine *Streptomyces* sp. Strain W007 was expressed in *Pichia pastoris* X-33. The growth curve showed that the maximum production of MAS1 (2.1 U/mL) was achieved at 72 h, when the cell density at 600 nm reached approximately (Yuan et al., 2016).

#### 2.1.2 Effect of physicochemical parameters on lipase production

**FIGURE 2:**
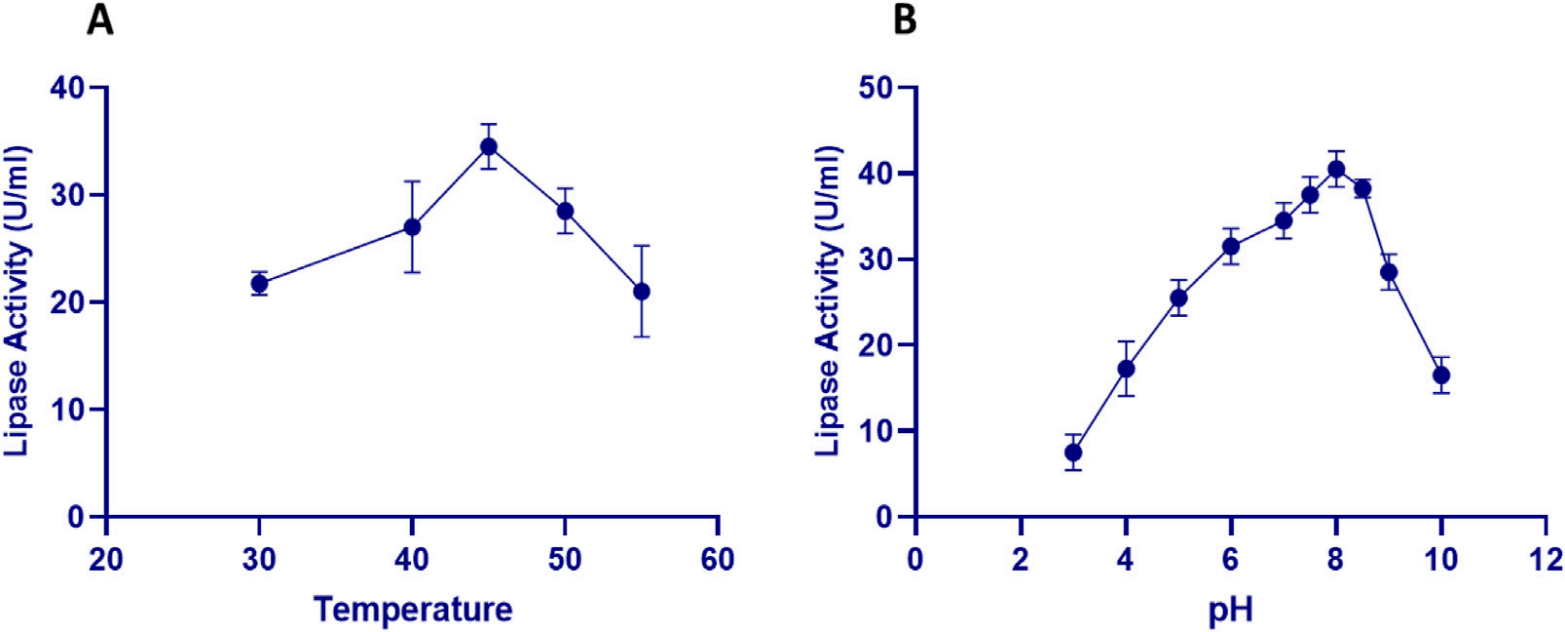
Variation in lipase activity produced by *S. gobitricini* at different temperatures **(A)** and pH values **(B)** using the basal medium containing 1% yeast extract and 1% glucose. Results were presented as mean ± SD from three independent experiments.

To identify the favorable conditions for extracellular lipase production, key factors such as pH, temperature, and agitation speed were examined. These parameters are crucial for influencing enzyme yield and activity. Figure 2A illustrates the effect of temperature on lipase activity from *S. gobitricini*. At 30°C, activity was 21.75 ± 1.06 U/mL, increasing to 27 ± 4.24 U/mL at 40°C. The highest activity (34.5 ± 2.12 U/mL) occurred at 45°C, indicating a maximal temperature. This aligns with typical enzymatic behavior, where increased temperature enhances molecular motion and reaction rates (Iyer and Ananthanarayan, 2008; Yuan et al., 1999). However, activity declined at 50°C (28.5 ± 2.12 U/mL) and 55°C (21 ± 4.24 U/mL), suggesting enzyme denaturation. The ability of *S. gobitricini* to maintain extracellular lipase activity at elevated temperatures highlights its adaptation to extreme conditions.

Lipase production by *S. gobitricini* varied with pH, showing a clear correlation between pH and enzyme functionality (Figure 2B). At pH 3.0, activity was low (7.5 ± 2.12 U/mL), indicating inhibition in acidic conditions. Activity increased with pH, peaking at 40.5 ± 2.12 U/mL at pH 8.0, suggesting performance in a slightly alkaline environment. Beyond this, activity declined, reaching 16.5 ± 2.12 U/mL at pH 10.0, highlighting reduced stability in highly alkaline conditions (Iyer and Ananthanarayan, 2008). *Streptomyces* adaptability to pH variations may be linked to physiological and biochemical mechanisms, including secondary metabolite production, intracellular pH regulation, and extracellular enzyme secretion (Sousa and Olivares, 2016). Similar trends were observed in *Streptomyces* strains like *Streptomyces sp*. Al-Dhabi-49 (pH 8.0, 168 ± 7.8 U/mL) (Al-Dhabi et al., 2020), *S. clavuligerus* (pH 6.8) (Dos Santos et al., 2017), and *S. exfoliatus* LP10 (pH 7.0) (Magda et al., 2012).

#### 2.1.3 Carbone and nitrogen sources

**FIGURE 3:**
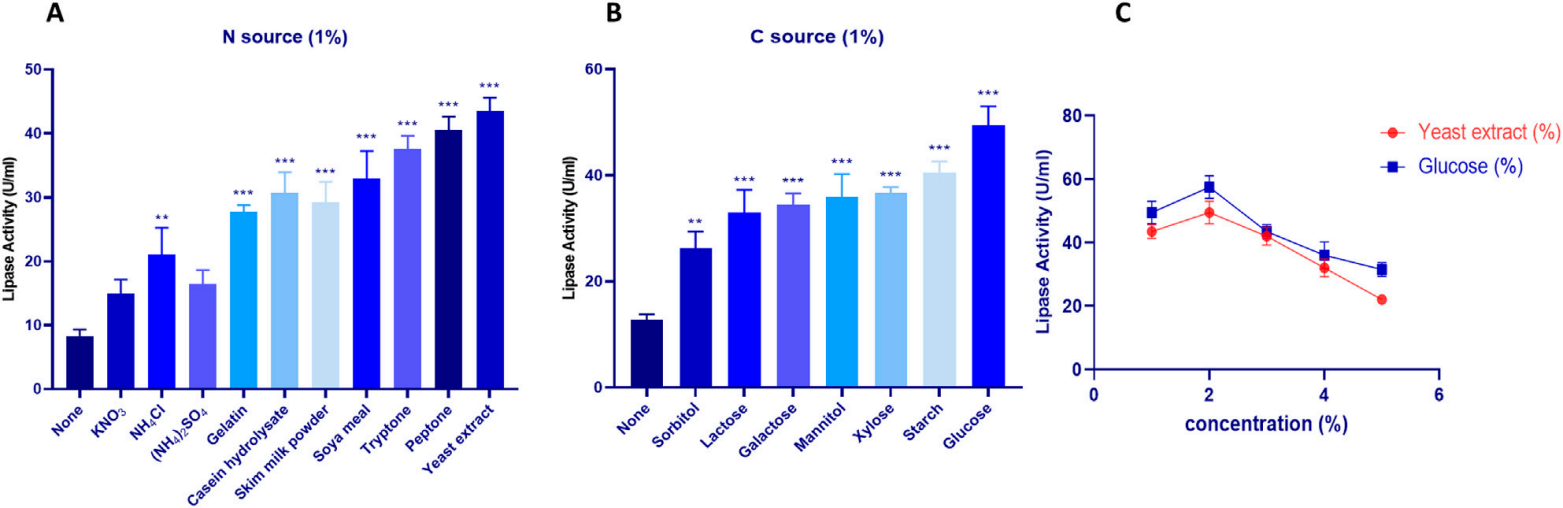
Impact of various Carbone sources at a concentration of 1% **(A)** and Nitrogen sources at a concentration 1% **(B)** on S*. gobitricini* lipase production. **(C)** Variation of lipase production with different concentrations of the selected Carbone source (Glucose) and Nitrogen source (Yeast extract). Results were presented as mean ± SD from three independent experiments, with asterisks indicating levels of significance (e.g., *p < 0.05; **p < 0.01; ***p < 0.001) above the bars in graphical representations.

Due to the significant importance of carbon catabolism in fermentation, the classical method ‘one variable at a time’ was adopted to enhance Lip_S.g_ production. The impact of carbon sources on lipase production by *S. gobitricini* is shown in Figure 3A. Without an added carbon source, activity was low (12.75 ± 1.06 U/mL), confirming the necessity of an external carbon source. Sorbitol moderately increased activity (26.25 ± 3.18 U/mL), while lactose and galactose further enhanced it (33 ± 4.24 and 34.5 ± 2.12 U/mL). Mannitol and xylose yielded similar levels (36 ± 4.24 and 36.75 ± 1.06 U/mL), with starch promoting higher activity (40.5 ± 2.12 U/mL). Glucose was the most effective, achieving 49.5 ± 3.53 U/mL. These findings align with studies on carbon source influence in *Streptomyces* strains like *S. tanashiensis* A2D, S*. griseocarneus*, and *S. padanus* PMS-702 (Ni et al., 2021). Glucose metabolism, regulated by glucokinase, plays a key role in enzyme production and alternative carbon utilization in *Streptomyces* (Diana et al., 2021). Lip_S.g_ activity peaked at 57.5 ± 3.53 U/mL at 2% glucose but declined at higher concentrations (Figure 3C), likely due to carbon catabolite repression (CCR), which regulates secondary metabolism and bioactive compound biosynthesis in *Streptomyces* (Lertcanawanichakul and Sahabuddeen, 2023).

Nitrogen is essential for metabolism and enzyme synthesis, influencing microbial growth and biochemical pathways (Blieva et al., 2021). Figure 3B shows the effect of nitrogen sources on lipase production by *S. gobitricini*. Without nitrogen, activity was low (8.25 ± 1.06 U/mL), confirming its necessity. Among inorganic sources, KNO_3_ led to moderate activity (15 ± 2.12 U/mL), while NH_4_Cl and (NH_4_)_2_SO_4_ produced 21 ± 4.24 and 16.5 ± 2.12 U/mL, respectively. Organic nitrogen sources significantly enhanced production, with gelatin (27.75 ± 1.06 U/mL) and casein hydrolysate (30.75 ± 3.18 U/mL). Higher activities were observed with skim milk (29.25 ± 3.18 U/mL) and soya meal (33 ± 4.24 U/mL), while tryptone (37.5 ± 2.12 U/mL), peptone (40.5 ± 2.12 U/mL), and yeast extract (43.5 ± 2.12 U/mL) yielded the highest activity. Yeast extract at 2% further enhanced production (Figure 3C), likely due to its rich composition of amino acids, vitamins, and growth factors that promote enzyme biosynthesis (Im et al., 2021). Similar trends were reported in *Streptomyces* strains, where glucose and malt extract enhanced lipase production in *Streptomyces* sp. Al-Dhabi-49 (Al-Dhabi et al., 2020), while NaNO_3_, tryptone, and peptone were the most effective for *Streptomyces* sp. TEM 33 (Cadirci et al., 2016).

#### 2.1.4 Triglycerides, detergents and metallic ions

The primary determinant factor for bacterial lipase expression has consistently been the Carbon source, as lipases are typically inducible enzymes activated by lipid-based substrates, such as oils or triglycerides, fatty acids, hydrolysable esters, and tweens (Gupta et al., 2004). The impact of various triglycerides and detergents on lipase production by *S. gobitricini* was investigated to evaluate their effect on enzyme activity and substrate availability in the culture medium. Table 1 shows that triglycerides and detergents significantly influenced lipase production. Olive oil induced the highest activity (107.5 ± 4.94 U/mL), likely due to its favorable fatty acid profile, followed by soybean and sunflower oils. Tributyrin and trioctanoin had moderate effects. Among detergents, Tween 80 significantly boosted activity (126.5 ± 3.53 U/mL), likely by enhancing substrate emulsification, while Triton X-100 had a lesser effect, and Tween 20 slightly reduced production. These findings highlight the role of both triglycerides and detergents in maximal lipase production, likely by improving substrate availability (Gupta et al., 2004). Olive oil and Tween 80 were also identified as the most effective carbon sources in other *Streptomyces* strains, including *S. griseus* (117.88 U/mL) (Vishnupriya et al., 2010) and *Streptomyces* sp. TEM 33 (Cadirci et al., 2016). Maximum enzyme production (486.66 U/mL) was achieved using Tween 20 as a carbon source in soil-derived *Streptomyces* sp. (Praveen Kumar et al., 2017).

**TABLE 1:** Effect of triglycerides, detergents and metallic ions (at 1% concentration) on *S. gobitricini* lipase production. Results were presented as mean ± SD from three independent experiments, Significant differences in lipase production compared to control group, with asterisks indicating levels of significance (e.g., *p < 0.05; **p < 0.01; ***p < 0.001).

Additives	Lipase activity (U/mL)
Triglycerides (1%)	
None	72.5 ± 3.53
Tributyrin	87 ± 4.24
Trioctanoin	87 ± 4.24
Olive oil	107.5 ± 4.94^***^
Soybean oil	95.5 ± 4.94^**^
Sunflower oil	90.5 ± 4.94^*^
Detergents (1%)	
None	106.5 ± 6.36
Tween 20	93 ± 4.24
Tween 80	126.5 ± 3.53^*^
Triton X-100	104.5 ± 6.36
Metal ions (1%)	
None	43.5 ± 2.12
BaCl_2_	48 ± 4.24
CaCl_2_	65.25 ± 3.18^***^
CoCl_2_	47 ± 2.82
MgCl_2_	43.5 ± 2.12
MgSO_4_	45.75 ± 3.18
FeCl_3_	36 ± 4.24
ZnCl_2_	42 ± 4.24

The effect of metal ions on lipase production by *S. gobitricini* showed that CaCl_2_ had the most significant impact, increasing activity to 65.25 ± 3.18 U/mL, suggesting its role in stabilizing enzyme structure and enhancing catalytic efficiency (Table 1). BaCl_2_ and CoCl_2_ also slightly increased activity (48 ± 4.24 and 47 ± 2.82 U/mL, respectively), while Mg^2+^, Zn^2+^, and Fe^3+^ had minimal or inhibitory effects possibly due to toxicity or interference with enzyme regulation. Metal ions likely influence lipase production by interacting with the enzyme’s active site, altering fatty acid solubility, and affecting bacterial metabolism. The enhancement observed with certain metal ions could be attributed to their ability to form complexes with ionized fatty acids in the medium, modifying their solubility and interfacial behavior. Additionally, metal ions play a role in bacterial metabolism, signaling pathways, and transport. While magnesium and zinc had little effect, Fe^3+^ significantly inhibited lipase production, potentially due to oxidative stress or disruption of essential cofactors required for enzyme expression (Ülker et al., 2011) (Hasan et al., 2006) (Zhang et al., 2012). In *Streptomyces* sp. Al-Dhabi-49, 0.1% Mg^2+^ increased lipase production to 163.7 ± 6.2 U/mL, whereas Hg^2+^ drastically reduced it (Al-Dhabi et al., 2020).

Beyond improving lipase production by *S. gobitricini*, the choice of fermentation method is crucial for ensuring scalability and efficiency. Two key approaches for microbial lipase production-submerged fermentation (SmF) and solid-state fermentation (SSF)-utilize agro-industrial residues as substrates. SmF, widely adopted in industrial applications, operates in a liquid medium, facilitating enzyme recovery and allowing precise control over critical parameters such as pH, temperature, and oxygen levels. In contrast, SSF, which requires minimal water, offers higher productivity and environmental benefits but presents engineering challenges at larger scales, such as maintaining uniform temperature and moisture gradients (Szymczak et al., 2021). Further studies are needed to enhance the scale-up of *S. gobitricini* production, which typically begins in bench-scale bioreactors before transitioning to larger systems for commercial use. The primary objective of scaling up is to replicate and enhance the efficiency of small-scale findings in larger bioreactors. Successful scale-up strategies depend on key parameters, including the volumetric oxygen transfer coefficient, volumetric power consumption, impeller tip speed, and mixing time.

### 2.2 Lip_S.g_ purification

**FIGURE 4:**
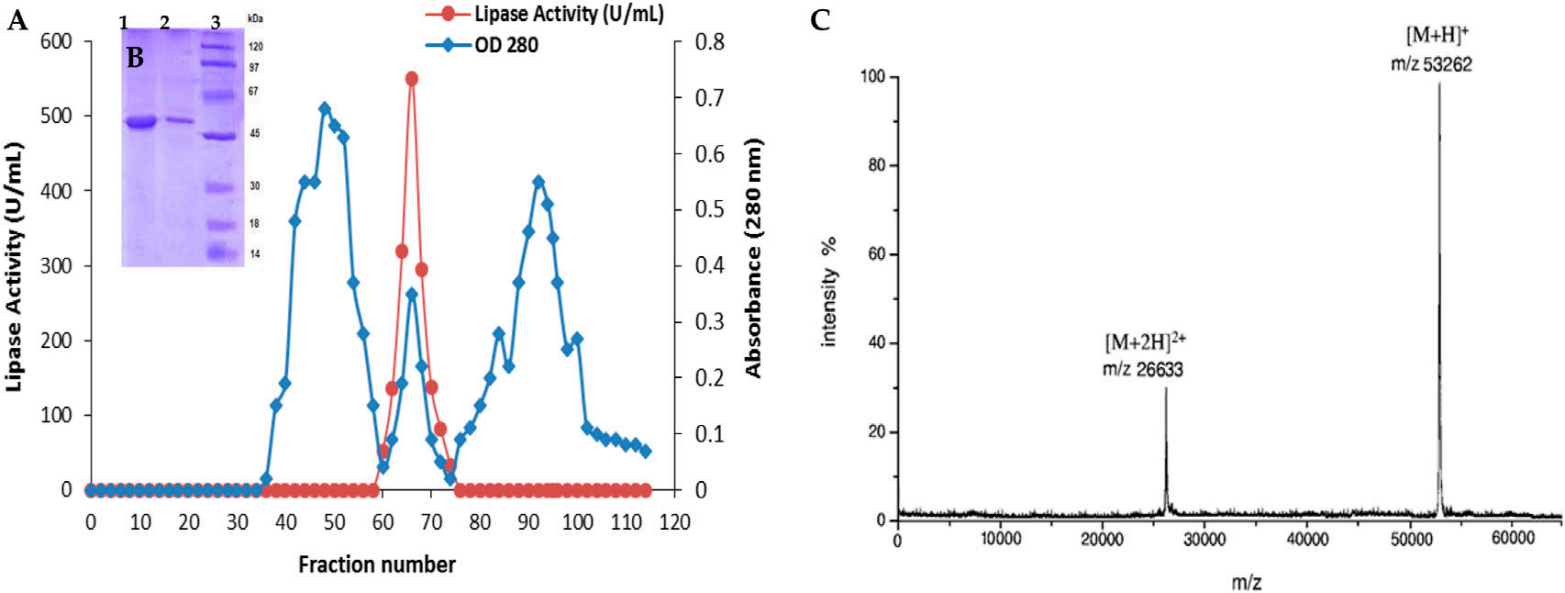
Chromatography profile on Sephadex S-200 column **(A)**, SDS-PAGE analysis **(B)** and MALDITOF analysis **(C)** of the purified Lip_S.g_. Figure 4B: Lane 1 and 2: purified Lip_S.g_ (5 and 10 μg, respectively). Line 3: molecular mass markers.

Lip_S.g_ was purified according to the procedure outlined in the materials and methods section. Initially, 250 mL of cultured supernatant was heat-treated at 70°C for 15 min. This was followed by fractionation using ammonium sulfate ((NH_4_)_2_SO_4_) at concentrations ranging from 25% to 65% (w/v). The resulting sample was then applied to a Sephadex S-200 column for further separation. Fractions exhibiting lipase activity, determined under standard assay conditions (Figure 4A) using olive oil as the substrate, were analyzed by SDS-PAGE. This revealed a single band with an approximate molecular weight of 50 kDa (Figure 4B). The molecular weight of the purified Lip_S.g_ was further confirmed by MALDI-TOF mass spectrometry, which determined a molecular mass of 53,262 Da (Figure 4C). Table 2 summarized the specific activity (SA) and recovery rates for Lip_S.g_ at each purification stage. The process achieved a recovery rate of 24.13% and a purification factor of 11.48. The specific activity of the purified lipase was 1750 U/mg under standard assay conditions. This result was consistent with other lipases purified from *Streptomyces* strains. For instance, a partially purified lipase from soil-derived *Streptomyces sp*. Exhibited a specific activity of 172.04 U/mg, a purification factor of 2.03, and a molecular weight of 45 kDa, as determined by SDS-PAGE analysis (Praveen Kumar et al., 2017). An organic solvent-tolerant lipase from *Streptomyces sp*. CS133 showed a 1.8-fold purification factor, with an estimated molecular mass of 39.8 kDa, also determined by SDS-PAGE (Zhang et al., 2012). In contrast, other studies reported lipases from *Streptomyces* strains with smaller molecular masses. For example, lipases from *Streptomyces sp*. CS326 and *marine Streptomyces sp*. W007 were found to migrate as single protein bands corresponding to molecular masses of 17 kDa and 29 kDa, respectively (Cho et al., 2012; Yuan et al., 1999).

**TABLE 2:** Purification steps of Lip_S.g_.

Purification Step	Total activity (U)	Protein (mg)	Specific activity (U/mg)	Activity Recovery (%)	Purification factor
Culture Supernatant (250 mL)	31,250	205	152.44	100	1
Heat treatment (70°C, 15 min)	21,875	72	303.82	70	2
(NH_4_)_2_SO_4_ Precipitation (25%–65%)	14,218	13.70	1,037.81	45.50	6.81
Sephadex S-200	7,540	4.31	1750	24.13	11.48

While the purification of Lip_S.g_ was successfully achieved at the laboratory scale, scaling up the process introduces several technical and economic challenges that must be carefully addressed. In chromatography-based purification, scale-up is typically achieved by increasing the column diameter and volumetric flow rate while maintaining a constant media bed height and linear flow rate. This approach ensures consistent residence time across different scales, preserving separation efficiency. However, large-scale purification presents additional complexities, including buffer selection, media packing, column engineering, and process hygiene, all of which can impact commercial biopharmaceutical manufacturing. The choice of chromatography media is particularly critical, as it directly influences purity levels and downstream processing efficiency (Milne, 2017).

### 2.3 Lip_S.g_ biochemical characterization

#### 2.3.1 Effect of temperature and pH on Lip_S.g_ activity and stability

**FIGURE 5:**
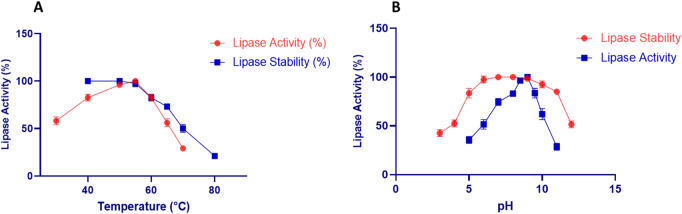
Impact of various temperatures **(A)** and pHs **(B)** on Lip_S.g_ activity and stability. Results were presented as mean ± SD from three independent experiments.

The purified Lip_S.g_ exhibited maximal activity at 50°C, with stability maintained between 40°C and 55°C but declining above 55°C due to thermal denaturation (Figure 5A). Its thermostability may be linked to structural factors such as hydrophobic clustering and salt-bridge density (Rabbani et al., 2023). Lip_S.g_ also showed highest activity at pH 9.0, with stability maintained from pH 5.0 to 10.0, but decreasing in extreme acidic or basic conditions (Figure 5B). These characteristics align with microbial lipases, such as *Streptomyces sp*. CS326 (pH 7.0, 40°C) (Cho et al., 2012), *S. bacillaris* (45°C, pH 9.0) (Gao et al., 2021), and lipase LS133 from *Streptomyces* sp. CS133 (stable at pH 5.0–9.0, maximal activity at pH 7.5, 40°C) (Mander et al., 2012). The purified Lip_S.g_ exhibited peak activity at 50°C, with stability maintained between 40°C and 55°C but declining above 55°C due to thermal denaturation (Figure 5A). Its thermostability may be linked to structural factors such as hydrophobic clustering and salt-bridge density (Rabbani et al., 2023). Lip_S.g_ also showed a highest activity at pH 9.0, with stability maintained from pH 5.0 to 10.0, but decreasing in extreme acidic or basic conditions (Figure 5B). These characteristics align with microbial lipases, such as *Streptomyces sp*. CS326 (maximal activity at pH 7.0, 40°C) (Cho et al., 2012), *S. bacillaris* (maximal activity at 45°C, pH 9.0) (Gao et al., 2021), and lipase LS133 from *Streptomyces sp*. CS133 (stable at pH 5.0–9.0, maximal activity at pH 7.5, 40°C) (Mander et al., 2012).

#### 2.3.2 Effect of calcium on Lip_S.g_ activity

**FIGURE 6:**
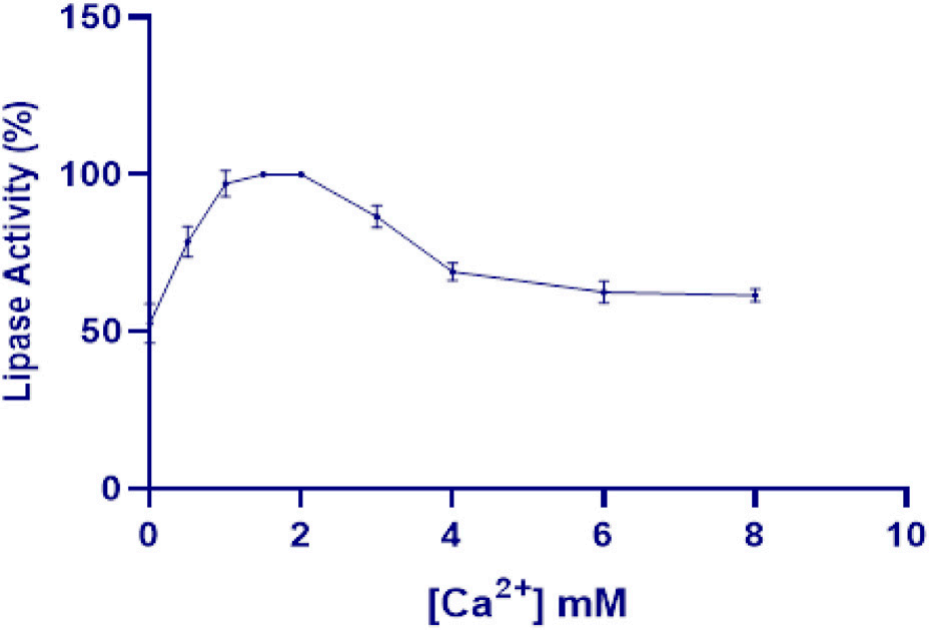
Effect of various concentrations of calcium on Lip_S.g_ activity. Results were presented as mean ± SD from three independent experiments.

While cofactors are generally not essential for lipase activity, divalent cations, particularly calcium, often enhance catalytic activity. This enhancement is attributed to increased interaction between calcium ions and long-chain fatty acids during hydrolysis, which facilitates the enzyme’s catalytic function (Gupta et al., 2004). In this context, the effect of calcium on Lip_S.g_ activity showed that increasing calcium concentrations activated the enzyme, with maximum activity (100%) observed at 1.5–2 mM (Figure 6). At lower concentrations or in total absence of calcium (0 and 0.5 mM), enzyme activity was significantly reduced, indicating that calcium is essential for highest functional performance. Moreover, metal ions like calcium are known to interact with specific pockets on the enzyme surface, stabilizing and maintaining the conformation of flexible segments of the polypeptide chain through coordination. This structural support further enhanced Lip_S.g_ activity (Shirazi et al., 2013). However, beyond 2 mM, enzyme activity started to decline, with a steady decrease observed at higher calcium concentrations (3–8 mM) (Figure 6).

Several *Streptomyces* lipases exhibit similar responses to calcium ions. For example, lipase from *S. bacillaris* displayed increased activity at calcium concentrations of 1 mM and 10 mM, reaching 116.2% ± 1.3% and 124.3% ± 2.7% activity, respectively (Gao et al., 2021). In contrast, lipase activities from *Streptomyces sp*. Strain W007 and *Streptomyces sp*. CS133 were unaffected by calcium at 1 mM, retaining 100% activity regardless of calcium presence (Mander et al., 2012; Yuan et al., 1999).

#### 2.3.3 Lip_S.g_ substrate specificity and apparent kinetic parameters determination

The substrate specificities of microbial lipases, especially regarding fatty acid and positional preferences, are crucial for predicting their catalytic activity and selecting specific lipases for targeted reactions, making them highly relevant for various applications (Song et al., 2008). The substrate specificity of purified Lip_S.g_ was assessed using a range of synthetic and natural substrates (Table 3). Data in Table 3 indicated that Lip_S.g_ showed the highest activity with TC18 (100%, followed closely by TC4 (92% ± 2.82%) and TC8 (91% ± 4.24%), suggesting a preference for long chain triglycerides. Among the tested oils, coconut and sesame oils exhibited high activity (88% ± 4.24% and 87% ± 4.24%, respectively), while sunflower oil showed the lowest (77% ± 2.82%). This could be attributed to their polyunsaturated fatty acid content, particularly oleic acid (C18:1 ω9) (Bacha et al., 2019). Lip_S.g_’s substrate preference may be due to specific enzyme-substrate interactions, influenced by the acyl-binding site’s hydrophobic residues (Albayati et al., 2020). Similar selectivity has been observed in *Streptomyces* lipases, such as *Streptomyces* sp. CS326, which preferred long-chain fatty acids (C16) (Cho et al., 2012), *S. bacillaris*, which showed maximal activity with C16 and significant activity with C14 and C12 (Gao et al., 2021), and *Streptomyces sp*. CS133, which favored C10–C18 substrates (Mander et al., 2012)*. Streptomyces sp*. W007 hydrolyzed esters from C4 to C18, with a preference for C8 (Yuan et al., 1999).

**TABLE 3:** Substrate specificity and apparent kinetic parameters of purified Lip_S.g_. Results were presented as mean ± SD from three independent experiments Significant differences in lipase activity compared to TC18, with asterisks indicating levels of significance (e.g., *p < 0.05; **p < 0.01; ***p < 0.001).

Substrate specificity	
Substrate	Lipase activity (%)
TC4	92 ± 2.82
TC8	91 ± 4.24
TC18	100 ± 0
Coconut oil	88 ± 4.24
Sesame oil	87 ± 4.24^*^
Soybean oil	84.5 ± 4.94^*^
Sunflower oil	77 ± 2.82^**^

Apparent kinetic parameters			
	TC4	TC8	TC18
V_max_ (µmol. min^-1^. mg^-1^)	2500 ± 0		
K_m_ (mM)	26.70 ± 0.063	13.22 ± 0.106	6.45 ± 0.289
k_cat_ (s^-1^)	2228.16 ± 0		
k_cat_/K_m_ (s^-1.^ mM^-1^)	83.43 ± 0.198	168.48 ± 1.351	345.53 ± 15.51

**FIGURE 7:**
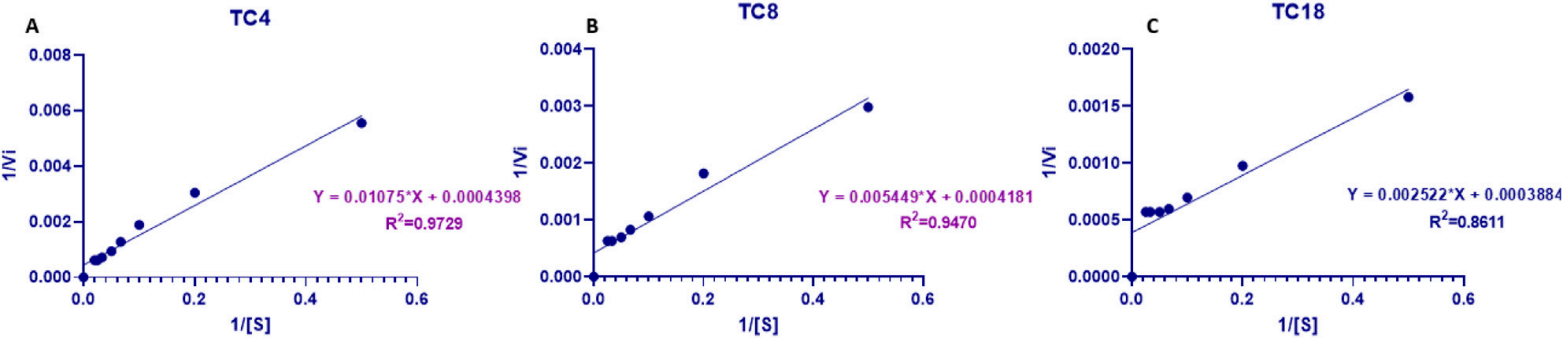
Lineweaver-Burk plots of Lip_S.g_ at varying substrate concentrations: **(A)** TC4, **(B)** TC8, and **(C)** TC18.

Kinetic analysis of purified Lip_S.g_ using Lineweaver–Burk plots (Figure 7) revealed a maximum reaction rate (V_max_ = 2500 μmol min^-1^·mg^-1^) and a turnover number (kcat 2228.16 s^-1^) across all substrates. The enzyme exhibited varying kinetic behaviors toward different triglycerides. Notably, the lowest apparent K_m_ was observed for TC18 (6.45 mM) and the highest for TC_4_ (26.70 mM), while the catalytic efficiency (k_cat_/K_m_) increased with chain length-ranging from 83.43 s^-1^ mM^-1^ for TC_4_ to 345.53 s^-1^ mM^-1^ for TC_18_. While K_m_ is sometimes interpreted as an inverse measure of enzyme-substrate affinity, this is only valid under strict Michaelis–Menten assumptions such as rapid equilibrium between Enzyme and Enzyme-Substrate and no allosteric or cooperative behavior. More precisely, K_m_ represents the substrate concentration at which the reaction rate is half of V_max_, and its value reflects a combination of binding and catalytic rate constants. Therefore, in this study, differences in K_m_ values likely reflect both substrate binding affinity and catalytic steps, influenced by the structural compatibility of each triglyceride with the enzyme’s active site. Kinetic parameters of various *Streptomyces* lipases show considerable variation, likely due to differences in substrates and methods used to measure lipase activity. For example, the lipase from *Streptomyces* sp. CS326, using pNPP as a substrate, displayed K_m_ and V_max_ values of 0.24 mM and 4.6 Mm min^-1^·mg^-1^, respectively (Cho et al., 2012).

### 2.4 Lip_S.g_ biotechnological applications

#### 2.4.1 Detergence application

Enzyme stability in the presence of surfactants and oxidizing agents is essential for applications in detergent formulations (Alonazi, 2024). For effective washing performance, a detergent lipase must be compatible with and stable in commonly used detergent components such as surfactants, bleaches, oxidizing agents, and other additives (Sarac et al., 2015). Thus, the stability of Lip_S.g_, compared to SAL4 and Lipolase, was assessed against various surfactants, oxidizing agents, boron compounds, and enzyme inhibitors (Table 4). Lip_S.g_ exhibited the highest stability across all surfactants, maintaining or enhancing its activity, particularly in sodium cholate (110% ± 5%), likely due to enhanced electrostatic interactions with the protein (Delorme et al., 2011). It also showed strong performance in Triton X-100 (105% ± 6.24%) and Tween 80 (105% ± 5%), where hydrophobic interactions played a significant role (Delorme et al., 2011) (Table 4). Similarly, SAL4 demonstrated activity above 100% in most surfactants, with notable stability in sodium cholate (107.33% ± 2.51%) and Triton X-100 (105.66% ± 1.15%). These results were consistent with structural studies suggesting that surfactants can improve substrate availability by emulsifying, forming mixed micelles, and inducing lipase conformational changes that increase active site accessibility (Delorme et al., 2011). In contrast, Lipolase displayed a marked decline in Triton X-100 (49.66% ± 4.16%) compared to sodium taurocholate (80.16% ± 2.02%) and sodium cholate (87.9% ± 3.15%) (Table 4), likely due to surfactant interactions that reduce lipolytic efficiency by forming inactive enzyme-surfactant complexes or impairing substrate binding at the interface (Delorme et al., 2011). This highlighted the importance of carefully evaluating surfactant effects, as they could either enhance or inhibit lipase activity, depending on their type. Among purified *Streptomyces* lipases, only the lipase from marine *Streptomyces sp*. Strain W007 has been tested against various surfactants. This enzyme showed reduced activity in dioctyl sulfosuccinate and SDS but increased to 133.91% after 2 h-incubation in N-lauroyl sarcosine sodium. Zwitterionic surfactants like soy lecithin and sulfopropyl betaine also decreased activity significantly. Nonionic detergents such as Tween-20, −60, −80, Triton X-100, and nonylphenol ethoxylates inhibited this enzyme, although it retained 84.5% activity with polyethylene oxide lauryl ether (Yuan et al., 1999).

**TABLE 4:** Biotechnological applications of purified Lip_S.g:_ stability in surfactant, oxidizing agent, boron compounds and enzymes inhibitors. SAL4 and Lipolase were used as positive controls for a comparative study. Results were presented as mean ± SD from three independent experiments, with asterisks indicating levels of significance (e.g., *p < 0.05; **p < 0.01; ***p < 0.001).

Enzymes	LipS.g		SAL4	Lipolase
Surfactant				
Control	100 ± 0		100 ± 0	100 ± 0
Sodium taurocholate	100 ± 4		103 ± 1.73	80.16 ± 2.02***
Sodium cholate	110 ± 5		107.33 ± 2.51	87.9 ± 3.15***
Triton X-100	105 ± 6.24		105.66 ± 1.15	49.66 ± 4.16***
Tween 80	105 ± 5		100.66 ± 2.08	72.71 ± 2.34***
Tween 20	105.33 ± 5.03		102.33 ± 2.51	82.36 ± 1.64***
Oxidizing agent				
Control	100 ± 0	100 ± 0	100 ± 24.51	
H2O2	76.66 ± 4.16	89.33 ± 4.04***	55.33 ± 5.27***	
Sodium hypochloride	64.66 ± 5.03	78 ± 3***	51 ± 6.46***	
Sodium perborate	73.33 ± 4.72	91 ± 3.06***	61.33 ± 14.65***	
Boron compounds				
Control	100 ± 0	100 ± 0	100 ± 0	
BKO2	108.33 ± 3.51	105 ± 2	78 ± 4***	
H3BO3	81 ± 3.60	89 ± 3*	69 ± 3***	
NaBO2	103 ± 3.60	107.66 ± 2.51	84 ± 4***	
Na2B4O7	106.66 ± 3.60	103.33 ± 3.51	97.33 ± 2.51**	
Enzyme inhibitors				
Control	100 ± 0	100 ± 0	100 ± 0	
SDS	89.33 ± 2.51	88 ± 3.60	72.33 ± 2.51***	
PMSF	98 ± 6.08	90.66 ± 4.04*	77.33 ± 2.51***	
EDTA	84.33 ± 5.03	81.33 ± 5.68	64.33 ± 4.72***	

When exposed to oxidizing agents, SAL4 exhibited the highest resistance, maintaining substantial activity in hydrogen peroxide (89.33% ± 4.04%) and sodium perborate (91% ± 3.06%). In contrast, Lipolase experienced significant reductions, particularly in sodium hypochlorite (51% ± 6.46%) and hydrogen peroxide (55.33% ± 5.27%) (Table 4). Among the boron compounds tested, Lip_S.g_ demonstrated the best stability, especially in BKO_2_ (108.33% ± 3.51%) and Na_2_B_4_O_7_ (106.66 %± 3.60%), while Lipolase showed the weakest performance, particularly in BKO_2_ (78% ± 4%) and H_3_BO_3_ (69% ± 3%) (Table 4).

The use of boron-based compounds is particularly relevant because they are commonly found in detergents and industrial formulations, where lipases are frequently applied. Testing enzyme stability in their presence helps to assess the potential compatibility and robustness of lipases under real industrial conditions. Furthermore, boron compounds such as sodium perborate and borax can act as mild oxidants or buffer agents, making them valuable tools to evaluate enzyme resilience to oxidative and ionic stress (Sarac et al., 2015).

Hydrogen peroxide is known to oxidize surface-exposed residues such as methionine, cysteine, tyrosine, and tryptophan in proteins, which can lead to a reduction in enzymatic activity. According to Törnvall et al. (2010), SAL4 and Lip_S.g_ seemed to have fewer oxidation-sensitive amino acids compared to Lipolase. Notably, all cysteine residues in SAL4 and Lip_S.g_ are likely involved in disulfide bonds, which are more resistant to oxidation than free cysteines. Oxidation can also alter the net charge and induce changes in the secondary and tertiary structures of enzymes (Törnvall et al., 2010).

In the presence of enzyme inhibitors, both Lip_S.g_ and SAL4 demonstrated strong resistance, retaining over 80% activity in SDS, PMSF, and EDTA. In contrast, Lipolase was more affected, particularly by SDS (72.33% ± 2.51%) and EDTA (64.33% ± 4.72%) (Table 4). The observed stability of Lip_S.g_ in the presence of PMSF, a well-known serine inhibitor, could be attributed to the serine residue being buried within the enzyme’s hydrophobic core, making it less accessible to PMSF (Sharma et al., 2012). Similarly, the stability of Lip_S.g_ and SAL4 in the presence of EDTA could be linked to the inaccessibility of the calcium ions, which enhance lipase activity. While EDTA likely chelates these ions, the enzyme remains unaffected due to their reduced exposure (Invernizzi et al., 2009). Additionally, the stability of Lip_S.g_ in the presence of SDS may be explained by the formation of an SDS-lipase complex, which increases the hydrophobicity of the protein surface at low concentrations (Delorme et al., 2011).

Overall, Lip_S.g_ and SAL4 consistently outperformed Lipolase in all tested conditions, making them more suitable for applications requiring high stability in detergent formulations and resistance to environmental stressors. The enhanced stability of Lip_S.g_ in the presence of oxidizing agents, boron compounds, and enzyme inhibitors makes it an ideal candidate for incorporation into detergent formulations (Guncheva and Zhiryakova, 2011).

**FIGURE 8:**
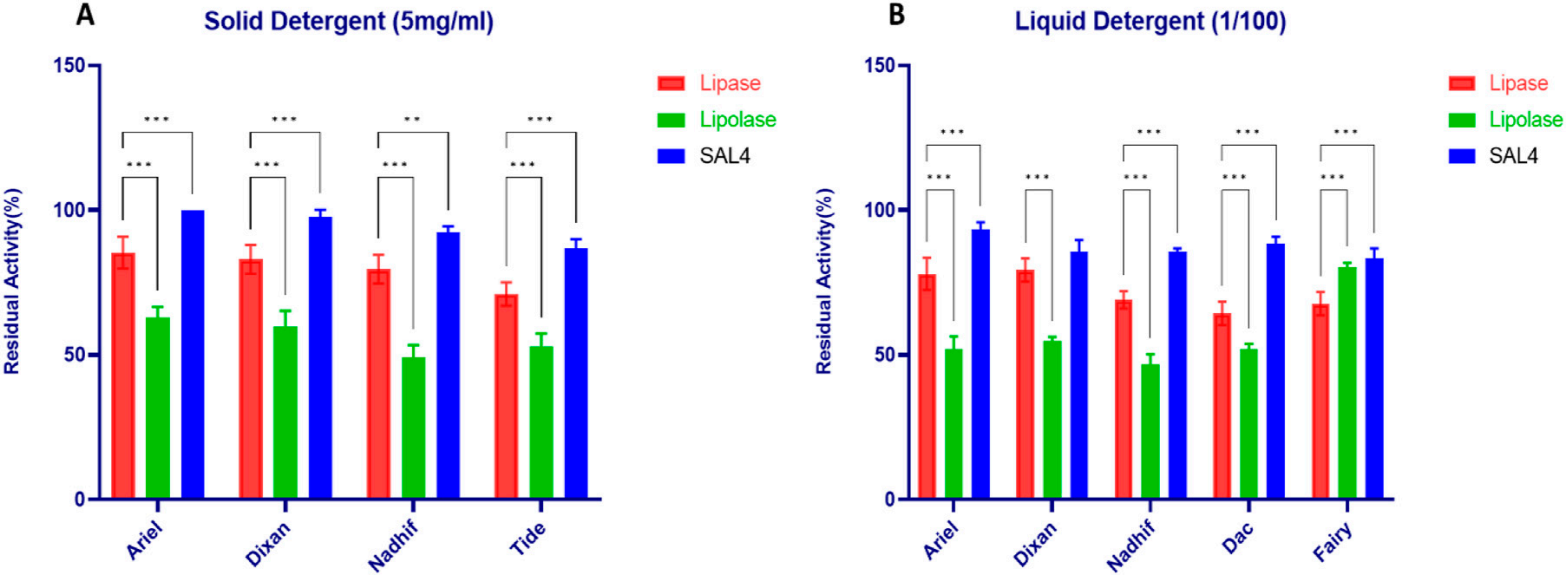
Stability of purified Lip_S.g_ in **(A)** Solid detergent at 5 mg/mL and **(B)** liquid detergent at 1/100 ratio. SAL4 and Lipolase were used as positive control to a comparative study. Results were presented as mean ± SD from three independent experiments, asterisks above bars indicate significant differences with the LipS.g (*p < 0.05; **p < 0.01; ***p < 0.001) (Dunnett test).

Figure 8 illustrated the stability of Lip_S.g_ in solid and liquid detergents at concentrations of 5 mg/mL (Figure 8A) and 1/100, respectively (Figure 8B), compared to SAL4 and Lipolase. The data showed that Lip_S.g_ exhibited moderate stability in solid detergents, with residual activity ranging from 71% ± 4% to 85.3% ± 5.5% (Figure 8A). However, SAL4 consistently outperformed Lip_S.g_, demonstrating superior stability with over 90% activity in most solid detergents, indicating its greater resistance to detergent components (Figure 8A). In contrast, Lipolase exhibited the lowest residual activity, dropping to around 50% in Nadhif and Tide, highlighting its significantly lower stability compared to both Lip_S.g_ and SAL4. In liquid detergents, SAL4 maintained the highest activity, with over 83% in all detergents, peaking at 93.33% ± 2.5% in Ariel (Figure 8B). Lip_S.g_’s activity ranged from 64.33% ± 4.04% in Dac to 79.33% ± 4.04% in Dixan, while Lipolase was the least stable, with residual activities ranging from 46.67% ± 3.51% in Nadhif to 80.33% ± 1.52% in Fairy (Figure 8B). Thus, Lip_S.g_ and SAL4 demonstrated greater stability than Lipolase, highlighting their resistance to the denaturing effects of detergents and their potential as strong candidates for detergent formulations requiring high enzyme stability.

Several studies have reported bacterial lipases with high stability in liquid and solid detergents, as well as in detergent compounds such as surfactants, oxidizing agents, and enzyme inhibitors. For example, lipase produced by *Pseudomonas helmanticensis* HS6 strain retained 40%–80% activity after 3 h-incubation with commercial detergents, underscoring its potential for detergent industry applications (Phukon et al., 2020). A lipase from *Bacillus subtilis* has demonstrated resistance to surfactants, oxidizing agents, and commercial detergents (Saraswat et al., 2017). Likewise, Geobacothermophilus FMR12 exhibited high lipolytic activity at 70°C and pH 9.0, proving effective in detergent applications. Common strains used in detergent formulations include *Bacillus* licheniformis, Geobacillus species, *Serratia marcescens* DEPTK21, *Bacillus* flexus XJU-1, *Bacillus* pumilus SG2, as well as *Staphylococcus* arlettae, *Bacillus* cepacia, *Pseudomonas* fluorescens, and *Candida* species (Yao et al., 2021).

#### 2.4.2 Lip_S.g_-catalyzed ester formation

Lipases, a class of serine hydrolases, catalyze reactions such as esterification, interesterification, and transesterification in non-aqueous media, including organic solvents and supercritical fluids, making them valuable for producing biofuels and other esters (Stergiou et al., 2013; Martin et al., 2019; Mandari and Devarai, 2022). Among these, lipase-catalyzed esterification reactions hold significant industrial importance (Stergiou et al., 2013; Gamayurova et al., 2021). To evaluate its ester synthesis capabilities, Lip_S.g_ was tested under various conditions and compared to SAL4, a known biocatalyst in esterification (Ben Bacha et al., 2016). As shown in Table 5, Lip_S.g_ demonstrated varying conversion efficiencies across different ester substrates, similar to SAL4. Notably, Lip_S.g_ effectively synthesized antioxidants like propyl gallate and tyrosol acetate, with conversion yields of 45% ± 3.21% and 35.5% ± 4.04%, respectively (Table 4). Additionally, Lip_S.g_ catalyzed the production of butyl acetate (a pineapple flavor compound) and ethyl oleate (a biofuel) with yields of 41% ± 2.08% and 48.33% ± 2.5%, respectively, compared to SAL4’s yields of 39.34% ± 3.6% for butyl acetate and 31% ± 1.5% for ethyl oleate. Moreover, Lip_S.g_ catalyzed the esterification of oleic acid with starch, producing starch oleate, a potential fully biodegradable thermoplastic, and esterified ricinoleic acid, achieving yields of 34% ± 3.05% and 42% ± 3.05% for starch oleate and ricinoleic esters, respectively (Table 4). These esterification reactions were carried out using the ethanol as alcohol. Starch oleate, a biodegradable and non-toxic sugar fatty acid ester, is hydrolyzed *in vivo* by pancreatic lipase. Its synthesis through lipase-catalyzed esterification under mild conditions offers an eco-friendly alternative to conventional chemical methods that rely on acyl chlorides and toxic solvents (Rodríguez-Alonso et al., 2023). The esters synthesized serve as versatile compounds commonly used as flavors, fragrances, and antioxidants, with extensive applications in the food, beverage, cosmetic, and pharmaceutical industries (Stergiou et al., 2013). Conventional ester synthesis methods are unsustainable and generate materials with low biodegradability. In contrast, lipases offer a greener alternative for both polymer synthesis and degradation, particularly in biomedical applications. Their enzymatic approach reduces environmental impact by minimizing waste, lowering risks, and utilizing renewable resources, making them a promising tool for advancing sustainable polymer chemistry (Freije García and García Liñares, 2024).

**TABLE 5:** Production of esters by Lip_S.g_ compared to SAL4. Results were presented as mean ± SD from three independent experiments, with asterisks indicating levels of significance (e.g., *p < 0.05; **p < 0.01; ***p < 0.001).

Enzymatic esterification yield (%)		
Enzymes Esters	Lip_S.g_	SAL4
Butyl acetate	41 ± 2.08	39.34 ± 3.60
Propyl gallate	45 ± 3.21	41.66 ± 5.50
Tyrosol acetate	35.5 ± 4.04	42.33 ± 4.04
Starch oleate	34 ± 3.05	50 ± 4.35^***^
Ricinoleic ester	42 ± 3.05	45 ± 2
Ethyl oleate	48.33 ± 2.51	31 ± 1.5^***^

## 3 Materials and methods

### 3.1 Materials and reagents

Chromatography material (Sephadex S-200), SDS-PAGE technique, pH-stat and rotary shaker were obtained from Bio-Rad (Hercules, CA, United States). Voyager DE-RP MALDI-TOF mass spectrometer was obtained from Biosystem, Framingham, MA, United States.

Chemicals were obtained from commercial sources. Glucose, galactose, mannitol, lactose, sorbitol, starch, or xylose, casein hydrolysate, gelatin, peptone, skim milk powder, soya meal, yeast extract, tryptone, ammonium nitrate (KNO_3_), ammonuim chloride (NH_4_Cl), or ammonium dihydogen phosphate ((NH_4_)_2_SO_4_)), surfactants (Tween 20, Tween 80, or Triton X-100), metal ions (BaCl_2_, CaCl_2_, COCl_2_, MgCl_2_, MgSO_4_, FeCl_3_, or ZnCl_2_), Ethylenediaminetetraacetic acid (EDTA), Sodium Dodecyl Sulfate (SDS), or Phenylmethylsulfonyl fluoride (PMSF), surfactants (NaDC, NaTDC, Triton X-100, Tween-80, or Tween-20), boron compounds (BKO_2_, H_3_BO_3_, NaBO_2_, or Na_2_B_4_O_7_) and oxidants (hydrogen peroxide (H_2_O_2_), sodium perborate or sodium hypochlorite), were purchased from Bio-Rad (Hercules, CA, United States).

Lipid compounds (olive oil, coconut oil, sesame oil, soybean oil, sunflower oil, trioctanoin (TC8), and triolein (C18) were purchased from Sigma Aldrich (St. Quentin-Fallavier, France). Commercial lipolase was from Novo Nordisk, Denmark. Various laundry detergents were used including Ariel (Procter and Gamble, Switzerland), Dixan (Henkel, Spain), Nadhif (Henkel-Alki, Tunisia), and Tide (Procter and Gamble, Saudi Arabia). Liquid detergents employed were DAC (Henkel, Saudi Arabia), Dixan (Henkel, Spain), Fairy (Modern Industries, Saudi Arabia), and Nadhif (Henkel-Alki, Tunisia).

### 3.2 Medium conditions and components for lipase production

*S. gobitricini* strain used for lipase production in this study has been identified and deposited in a culture collection under the designation FA-KSU 23, with the accession number PP708563. This strain was isolated from polluted mangrove soil, identified, and preserved in the Botany and Microbiology Department, College of Science, King Saud University (Riyadh, Saudi Arabia).

To prepare the *S. gobitricini* culture, 50 mL of Basal Broth medium (pH 7.0) was dispensed into 500 mL Erlenmeyer flasks. After sterilization, approximately 2 mL (4 × 106 CFU/mL) of bacterial suspension, which had been grown in nutrient broth at 30°C for 2 days, was inoculated into the flask and incubated at 30°C for 7 days on a rotary shaker at 270 g.

The determination of media components, including Carbon at a concentration 1% (glucose, galactose, mannitol, lactose, sorbitol, starch, or xylose), Nitrogen at a concentration of 1% (casein hydrolysate, gelatin, peptone, skim milk powder, soya meal, yeast extract, tryptone, KNO_3_, NH_4_Cl, or (NH_4_)_2_SO_4_), surfactants (Tween 20, Tween 80, or Triton X-100), lipid compounds (olive oil, soybean oil, sunflower oil, TC4, or TC8), and metal ions (BaCl_2_, CaCl_2_, COCl_2_, MgCl_2_, MgSO_4_, FeCl_3_, or ZnCl_2_), as well as culture conditions such as incubation time (0–176 h), temperature (30°C–55°C), pH (pH 3.0–10.0), and agitation speed (67–270 g), was conducted by replacing the components in the initial production media. Samples were collected and subjected to centrifugation (10 min at 9,000 g) with the supernatant used as the crude enzyme extract for lipase assays. Growth was assessed by measuring the optical density of the culture at 600 nm. All experiments were performed in triplicate under aseptic conditions, with results reflecting the averages of these three trials.

An improved medium incorporating the best sources of carbon, nitrogen, detergent, triglycerides, and metal ions for enhanced lipase production was then developed, and the bacteria were cultivated in this refined medium.

### 3.3 Purification of Lip_S.g_

Lipase from *S. gobitricini* was produced in the refined medium at 45°C over 84 h. Cells were removed via centrifugation (30 min at 9,000 g), and the resulting crude enzyme solution (250 mL, 31,250 Total Units (UT)) was incubated for 15 min at 70°C. Following rapid cooling and subsequent centrifugation for 30 min at 9,000 g, the supernatant (242 mL, 21,875UT) was fractionated using solid ammonium sulfate at 20%–65% saturation. The precipitate obtained post-centrifugation was resuspended in 25 mM Tris–HCl, pH 8.0, supplemented with 50 mM NaCl and 2 mM benzamidine (12 mL, 14,218UT), and loaded onto a Sephadex S-200 column (1.6 × 110 cm) pre-equilibrated with the same buffer. Every 6 min, 2.0 mL fractions were collected and analyzed for protein content and lipase activity. Fractions exhibiting high lipase activity were pooled (50 mL, 7,540 UT), concentrated, and stored at 4°C until further use.

### 3.4 Determination of lipase activity

Using a pH-stat at 50°C and pH 9.0, lipolytic activity was determined titrimetrically with substrates including an olive oil emulsion stabilized by 10% Gum Arabic and TC4 (Rathelot et al., 1981). Some assays were performed in the presence of different concentrations of Ca^2+^ (0–8 mM) at pH 9.0 and at 50°C. For kinetic studies, coconut oil, sesame oil, soybean oil, sunflower oil, trioctanoin (TC8), and triolein (C18) were also used to measure enzyme activity (Abdelkafi et al., 2009). Lipase activity was quantified in international units (U), defined as the production of 1 μmol of fatty acid per minute.

### 3.5 Protein analysis

Bradford protocol was used to assess the protein content (Bradford, 1976). The purified lipase was subjected to electrophoretic analysis using SDS-PAGE (15%) following the Laemmli method (Laemmli, 1970).

The molecular mass of the native protein was then accurately determined on a Voyager DE-RP MALDI-TOF mass spectrometer (Biosystem, Framingham, MA, United States). Mass spectra recorded in linear mode were externally calibrated with suitable standards and analyzed by the GRAMS/386 Software.

### 3.6 Lip_S.g_ characterization

#### 3.6.1 pH and temperature effects on Lip_S.g_ activity and stability

Lipase activity was evaluated in different buffers at 0.5 M across a large range of pHs (5.0–11.0) at a temperature of 50°C. The stability of lipase at different pHs was assessed by incubating the enzyme at pH values between 3.0 and 11.0 for 1 hour at room temperature. The residual lipase activity was assessed following centrifugation under the standard assay method. The highest temperature for purified lipase activity was established by conducting enzyme assays across a range of temperatures (30°C–70°C) at pH 9.0. The thermal stability was assessed by incubating the lipase at pH 8.0 across a range of temperatures (40°C–80°C) and evaluating the residual activity after 1 h, following centrifugation, under standard titrimetric assay conditions. Each measurement was conducted on three separate assays.

#### 3.6.2 Kinetic study

Lipase activities were assessed as a function of different concentrations (0–40 mM) of various substrates (TC18, TC8 or TC4). The maximum velocity (V_max_) and the apparent Michaelis-Menten constant (K_m_app) for each reaction were determined by Lineweaver-Burk plot.

#### 3.6.3 Compatibility of Lip_S.g_ with oxidizing agents, surfactants, and commercial detergents

Lip_S.g_ was evaluated for its potential use in the detergent industry. 120 U sample of Lip_S.g_ (from the (NH_4_)_2_SO_4_ fraction) was incubated with various enzyme inhibitors (EDTA, SDS, or PMSF), surfactants (NaDC, NaTDC, Triton X-100, Tween-80, or Tween-20), boron compounds (BKO_2_, H_3_BO_3_, NaBO_2_, or Na_2_B_4_O_7_) and oxidants (hydrogen peroxide (H_2_O_2_), sodium perborate or sodium hypochlorite) at 1% for 60 min at 40°C.

Compatibility was also assessed with various commercial solid laundry detergents (Ariel (Procter and Gamble, Switzerland), Dixan (Henkel, Spain), Nadhif (Henkel-Alki, Tunisia), and Tide (Procter and Gamble, Saudi Arabia)) and liquid detergents (DAC (Henkel, Saudi Arabia), Dixan (Henkel, Spain), Fairy (Modern Industries, Saudi Arabia), and Nadhif (Henkel-Alki, Tunisia)) following the method of Cherif et al. (2011). Results were compared to commercial lipolase (Novo Nordisk, Denmark) and *Staphylococcus aureus* lipase (SAL4) from a previous study (Ben Bacha et al., 2018). All the experiments of characterization study were performed three times.

#### 3.6.4 Enzymatic esterification

The different esterification reactions were performed according to previously described protocols (Ghamgui et al., 2007). For comparison, SAL4 and commercial lipase were used as positive controls.

#### 3.6.5 Statistical analysis

All of the assays were done in biological triplicate with three technical replicates, and data were given as mean ± standard deviation (SD). The statistical analysis was carried out through one-way and two-way analysis of variance (ANOVA) as well as Duncan’s *post hoc* test using GraphPad Prism version 9 and Excel. A *p*-value of <0.05 was regarded as significant, and asterisks were used to indicate significance.

## 4 Conclusion

Lip_S.g_, a novel lipase from *S. gobitricini*, exhibited high enzyme activity under the best conditions that we assesed and remarkable stability in detergent formulations. Its efficiency in ester synthesis, including antioxidants and biofuels, highlighted its potential as a versatile biocatalyst. These properties suggested a wide range of industrial applications, notably in the food, beverage, cosmetics, and pharmaceutical sectors.

## Data Availability

The raw data supporting the conclusions of this article will be made available by the authors, without undue reservation.
